# Astrocytes Play a Key Role in *Drosophila* Mushroom Body Axon Pruning

**DOI:** 10.1371/journal.pone.0086178

**Published:** 2014-01-21

**Authors:** Yaniv Hakim, Shiri P. Yaniv, Oren Schuldiner

**Affiliations:** Department of Molecular Cell Biology, Weizmann Institute of Sciences, Rehovot, Israel; Alexander Fleming Biomedical Sciences Research Center, Greece

## Abstract

Axon pruning is an evolutionarily conserved strategy used to remodel neuronal connections during development. The *Drosophila* mushroom body (MB) undergoes neuronal remodeling in a highly stereotypical and tightly regulated manner, however many open questions remain. Although it has been previously shown that glia instruct pruning by secreting a TGF-β ligand, myoglianin, which primes MB neurons for fragmentation and also later engulf the axonal debris once fragmentation has been completed, which glia subtypes participate in these processes as well as the molecular details are unknown. Here we show that, unexpectedly, astrocytes are the major glial subtype that is responsible for the clearance of MB axon debris following fragmentation, even though they represent only a minority of glia in the MB area during remodeling. Furthermore, we show that astrocytes both promote fragmentation of MB axons as well as clear axonal debris and that this process is mediated by ecdysone signaling in the astrocytes themselves. In addition, we found that blocking the expression of the cell engulfment receptor Draper in astrocytes only affects axonal debris clearance. Thereby we uncoupled the function of astrocytes in promoting axon fragmentation to that of clearing axonal debris after fragmentation has been completed. Our study finds a novel role for astrocytes in the MB and suggests two separate pathways in which they affect developmental axon pruning.

## Introduction

Neuronal remodeling is an evolutionarily conserved process used to refine neuronal circuits during development in both vertebrates and invertebrates. One abundant type of neuronal remodeling is axon and dendrite pruning which involves the removal of exuberant connections in a tightly regulated process [1]. Axon pruning has been shown to share molecular similarities with axon degeneration following injury as well as ‘dying-back’ neurodegenerative diseases such as amyotrophic lateral sclerosis (ALS), spinal muscular atrophy (SMA) and the later stages of multiple sclerosis (MS) [1], [2]. Thus, uncovering the mechanisms of developmental axon pruning should shed light onto how axons are eliminated during development, disease and following injury.

**Figure 1:**
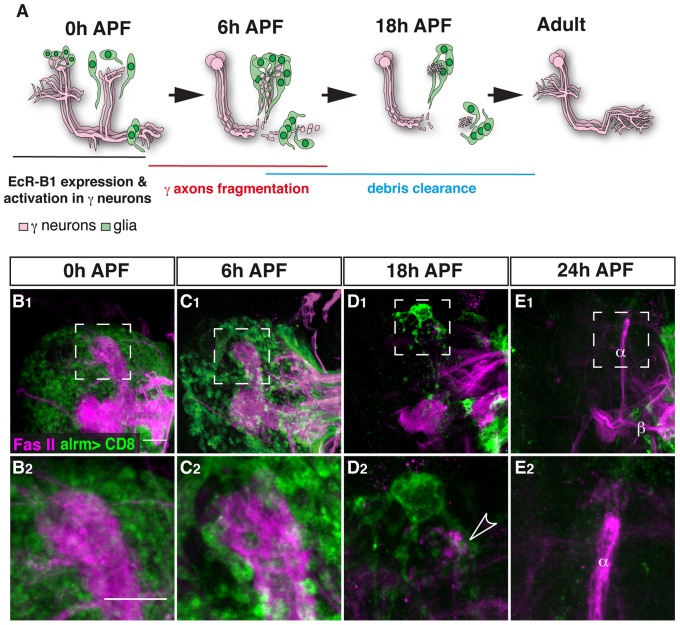
Astrocytes surround the MB during developmental axon pruning. (A) Scheme of developmental pruning of MB γ neurons. During larval development and up to puparium formation (0 h APF), γ neurons extend a single process that sends out dendrites near the cell body and continues as an axon peduncle that bifurcates to form a dorsal and a medial branch. At the onset of puparium, a glial derived TGF-β signal induces the expression of the ecdysone receptor-B1 (EcR-B1) within γ neurons. Subsequently, an ecdysone pulse activates EcR-B1, resulting in a largely unknown transcriptional cascade. At 6 h APF both axonal branches, as well as the dendrites begin to undergo fragmentation, while the pedunclar axon remains intact and the neurons retain their cell body. The fragmented axons are then cleared by glia that surround these axons. Subsequently, γ neurons regrow axons that project into a new, adult specific and medially projecting lobe. (B–E) Confocal Z-projections of brains expressing alrm-GAL4 driving mCD8::GFP at 0 h APF (B), 6 h APF (C), 18 h APF (D) or 24 h APF (E). (B) At the onset of metamorphosis (0 h APF) astrocytic membranes are evenly dispersed in the region of the MB lobes (higher magnification in A_2_). (C) By 6 h APF, at the onset of pruning, the astrocytes have changed their morphology and have begun to infiltrate the degenerating lobes. (D) At 18 h astrocyte membranes surround axon fragments (arrowhead in D_2_). By 18 h to (E) 24 h APF there is a significant decrease in alrm-GAL4 positive membranes. Newly extended axon branches of α/β neurons, are also stained with anti-FasII antibody at 24 h APF (E). Magenta represents anti-FasII staining. Green is alrm-GAL4 driven mCD8::GFP. The scale bars are 20 μm. Genotypes: (B–E) y,w;alrm-GAL4;alrm-GAL4,UAS-mCD8::GFP/+.

One highly attractive model of neuronal remodeling that has forwarded our understanding of the molecular and cellular mechanisms underlying these intricate processes is that of the *Drosophila melanogaster* mushroom body (MB) [3]. MB γ neurons undergo a highly stereotyped and tightly controlled process of dendrite and axon pruning (Figure 1A). During larval development and up to the onset of metamorphosis (0 h APF – after puparium formation), the early born MB γ neurons extend their axons to a medial and a dorsal lobe (Figure 1A). Axon fragmentation can be morphologically detected at around 6 h APF and is essentially completed by 18 h APF. At 24 h APF γ neurons start to regrow their axons to a new, adult specific lobe at the same time in which the later born α/β neurons begin to extend their axons. It appears that neighboring glia participate in the process in at least two distinct stages. Recently, glia were found to instruct axon fragmentation by secreting myoglianin (myo) [4], a TGF-β ligand, which binds to the TGF-β receptors baboon (babo) and punt/wishful thinking (put; wit) [5] assisted by the TGF-β accessory receptor plum [6] on the membrane of γ neurons. This in turn triggers the TGF-β pathway cell- autonomously within the MB γ neurons resulting in an increase in the expression of ecdysone receptor B1 (EcR-B1) in γ neurons [4], [5]. Since it has been shown that expression of EcR-B1 in γ neurons is essential, but not sufficient, for axon fragmentation [7], glial activity can be seen as ‘priming’ MB neurons for additional signals required for pruning. Additionally, it has been shown that once MB γ axons have begun to degenerate, glial cells complete the pruning process by engulfing and clearing the axonal debris [8], [9] in a process that is dependent on the glial expression of the apoptotic cell engulfment receptor Draper (Drpr) [10], [11].

Although much progress has been made in our understanding of the role of glia during neuronal remodeling of MB neurons, many open questions still remain. For example, little is known about the glial subtypes that participate in this process and their exact interaction with the neurons during pruning. While myo was found to be secreted mainly by cortex glia and, to a lesser extent, also by astrocytes [4], the relative contribution of each glial subtype in both the initiation of fragmentation as well as debris clearance is unknown. Recently, it has been shown that ensheathing glia are required for the engulfment of olfactory receptor (OR) axons undergoing Wallerian degeneration in a Drpr dependent manner [12]. Additionally, while it was shown that glia participate in the refinement of the neuromuscular junction in *Drosophila* [13] it is not known which glial type is involved. In mammals, microglia are the major phagocytic cell in the nervous system important for many processes including synaptic pruning [14]. It is therefore interesting to determine which glial subtypes play a role in pruning and what is their precise role/s. Where are they located? Are the same glial cells involved in secreting myo as those that are responsible for axon engulfment? How separate are these processes?

Here we report that astrocytes play several roles throughout MB remodeling and that they, not ensheathing glia, are the major glial subtype involved in clearing MB γ axon debris, despite the fact that they are very few in number. We show that blocking ecdysone receptor signaling in astrocytes reduces their ability to initiate fragmentation of γ axons as well as clear axonal debris. Furthermore, at the onset of axon pruning, astrocytes infiltrate the MB lobes and later on engulf most, if not all, of axonal debris in a process that is regulated, at least in part, by Drpr and endocytosis.

## Results

### Astrocytes infiltrate and engulf MB γ neurons during remodeling

Previous work has shown that glia play a major role in the clearance of MB γ axon fragments following developmental axon fragmentation [8], [9], however the precise identity of these glia is unknown. In the adult animal, injured olfactory receptor neurons undergoing Wallerian degeneration are engulfed by ensheathing glia [12]. As a first step, we first wished to identify which glia are located in the vicinity of the MB during metamorphosis. In order to do this, we labeled the two major types of neuropil glia using available glial subtype specific Gal4 drivers, alrm-Gal4 for astrocytes and mz0709-Gal4 for ensheathing glia [12]. We then followed these cells at the time points relevant for axon pruning.

We found that the ensheathing glia specific driver, mz0709-Gal4, is expressed in repo positive cells in the adult MB (arrows in Figure S1A) and in the adult antenna lobe (AL) (Figure S1C) as was previously reported [12]. However, this driver was not expressed in repo-positive cells at the axon pruning relevant time point of 6 h APF in either the vicinity of the MB (Figure S1B) or the AL (Figure S1D), indicating that, at least in these regions, this driver does not label glia at this developmental time point. This was surprising since it was already shown that ensheathing glia act as phagocytes in the adult fly brain [12]. New glial drivers that label ensheathing glia during metamorphosis are therefore necessary to study their role during remodeling.

In contrast, labeling astrocytes with alrm-Gal4 showed that astrocytes are adjacent to the γ lobe at 0 h APF and 6 h APF (Figure 1B,C) and even remained in the vicinity after axon fragmentation has been completed (Figure 1D). We also noticed a morphological change in the astrocytes throughout this developmental period. Between 0 h APF and 6 h APF astrocytic membranes became much more concentrated into discrete points that look like cysts, or beads on a string (compare Figure 1B_2_ to 1C_2_). Later, astrocytes gained “finger-like” extensions at 18 h APF (Figure 1D_2_) while by 24 h APF very few membranes were labeled (Figure 1E, see next section).

**Figure 2:**
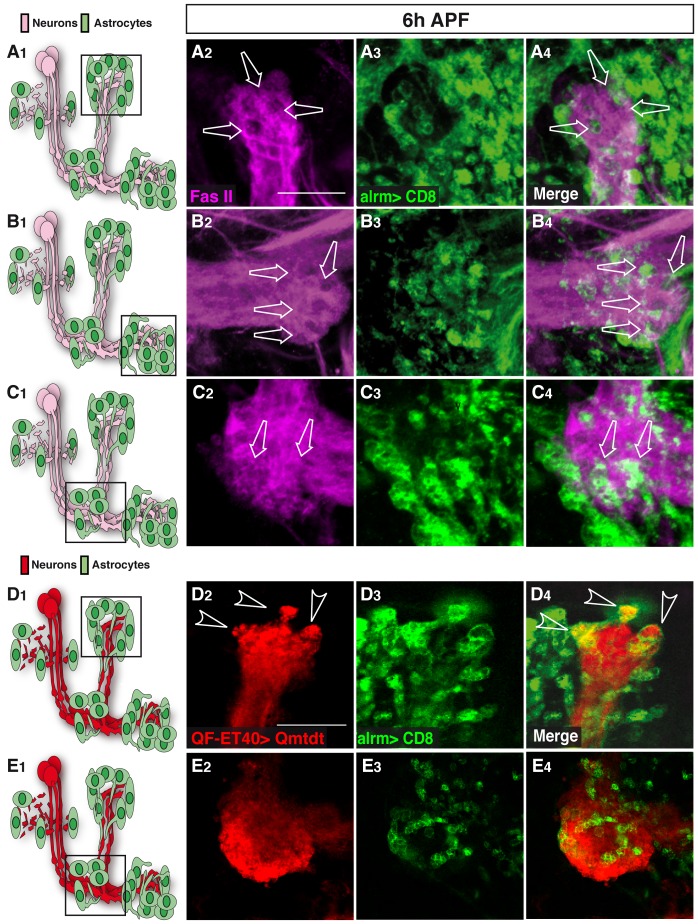
Astrocytes infiltrate and engulf degenerating MB γ neurons at 6h APF. (A–C) Confocal Z-projections of brains expressing alrm-GAL4 driven mCD8::GFP focusing on the MB medial (A) or dorsal (B) lobes or the peduncle (C) at 6 h APF as depicted by the cartoons on the left (A_1_–C_1_). Fas II staining shows clear spherical holes devoid of FasII expression in the MB γ dorsal (A_2_) and medial (B_2_) lobes and a slight decrease in FasII staining in the peduncle (C_3_). These holes are occupied by astrocyte extensions (A_3_–C_3_) infiltrating into the MB axon bundle (arrows in A_4_–C_4_). Magenta represents anti-FasII staining. Green is alrm-GAL4 driven mCD8::GFP. (D-E) Confocal single slices of the MB dorsal lobe tip (D) and the peduncle (E). While γ neurons were labeled by the Q-system (ET40-QF driving QUAS-mtdT-3xHA expression), astrocytes were labeled by alrm-GAL4 driven mCD8::GFP expression. At 6 h APF, γ axons are largely fragmented at the dorsal tip lobe (D_2_) but only few fragments are detected at the peduncle (E_2_). While astrocytes infiltrate both the dorsal lobe (D_4_) and the peduncle (E_4_), clear engulfment events can only be seen around the dorsal tip (D_4_). Red is QF-ET40 driven QUAS-mtdt-3xHA. Green is alrm-GAL4 driven mCD8::GFP. The scale bars are 20 μm. Genotypes: (A–C) w;alrm-GAL4;alrm-GAL4,UAS-mCD8::GFP/+ (D–E) y,w;QF-ET40,QUAS-mtdT-3xHA/+;alrm-GAL4,UAS-mCD8::GFP/+.

Upon closer analysis, we noticed that not only are the astrocytes in the vicinity of the γ lobes, but they actually infiltrate them (Figure 2). At 6 h APF FasII staining of γ axons shows clear spherical regions that are devoid of staining in the both the dorsal (arrows in Figure 2A_2_) and medial (arrows in Figure 2B_2_) γ lobes. While there appear to be areas in the peduncle where there is a slight decrease in FasII staining (arrows in Figure 2C_2_), this decrease is minor as compared to that which we observed in the medial and dorsal lobes so we believe it is not due to a significant amount of pruning. It appears that these low FasII regions are actually filled with extensions of astrocytes (Figure 2A_4_–2C_4_) suggesting that astrocytes not only infiltrate the lobes but also may actually engulf axonal material. However, to test this hypothesis we wanted to express a stable transgene in neurons and at the same time visualize the astrocytes. We thus decided to use two binary systems within the same animal, labeling neurons using the QF system (QF-ET40 driving QUAS-mtdt:HA) [15] and astrocytes using the GAL4 system (as previously, alrm-Gal4 driving UAS-mCD8-GFP). Indeed we found that the astrocytes were clearly engulfing axonal debris at the dorsal tip of the γ lobe at 6 h APF (Figure 2D). In fact, we found that most, if not all, axonal fragments were engulfed by astrocytic membranes. Interestingly, when we took a closer look at the peduncle area of the γ lobe we saw a different situation. This part of the axon peduncle does not undergo extensive fragmentation and indeed while we do see exploratory infiltration of astrocytes into this region, there is less active engulfment (Figure 2E). These results are consistent with previous findings that lysosomal activity is delayed in the axon peduncle compared to the lobes at this time point [8]. Unfortunately, the flies containing the dual binary systems were not healthy and very difficult to propagate and thus we performed all subsequent experiments by tracking FasII staining. Taken together, our results suggest that astrocytes, not ensheathing glia, are the main glial subtype that is responsible for the infiltration and engulfment of MB γ debris following axon fragmentation.

### The number of astrocytes at the MB stays constant throughout remodeling

**Figure 3:**
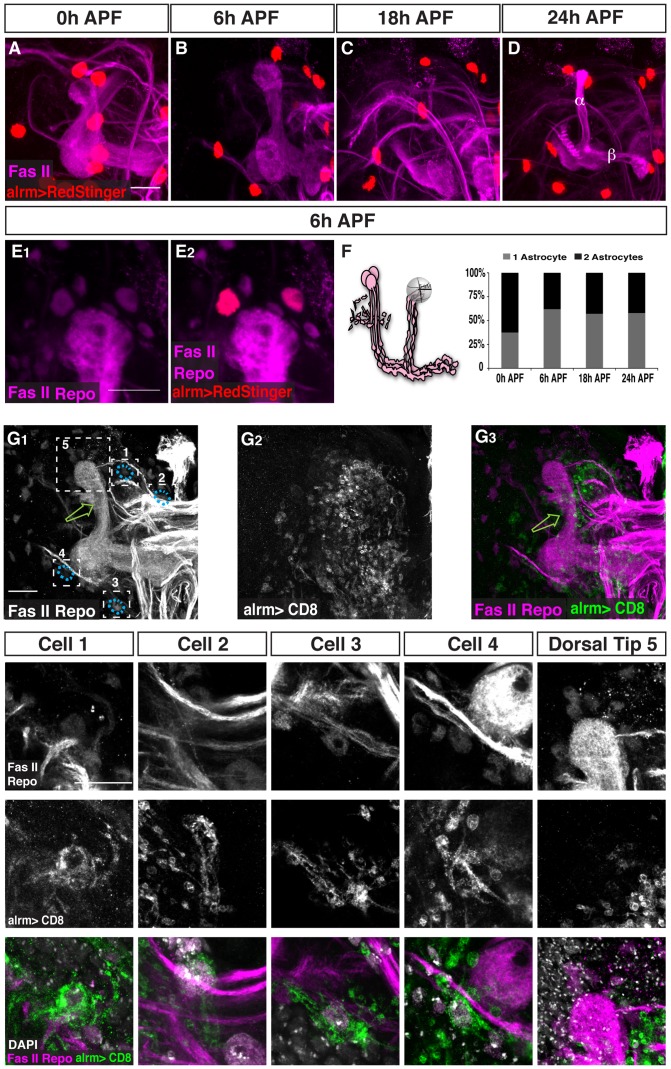
The number of astrocytes at the MB remains constant throughout remodeling. (A–E) Confocal Z-projections of alrm-GAL4 driven RedStinger expression (red, A–E) labeling astrocytes nuclei at 0 h APF (A), 6 h APF (B, E), 18 h APF (C) or 24 h APF (D). (A–D) Labeling astrocyte cell bodies shows that the number of the astrocytes does not significantly change during remodeling. (E) High magnification view of dorsal tip reveals that astrocytes (red) label only part of the repo^+^ nuclei in the vicinity of the MB lobe tip. (F) To characterize this quantitatively, we counted the number of astrocytes nuclei within 5 μM of the dorsal tip and found that only 1–2 astrocytes are located near the dorsal tip throughout remodeling. Magenta represents anti-FasII and anti-Repo staining. Red is alrm-GAL4 driven RedStinger. (G) Confocal Z-projections (G) or single slices (individual cells) of brains expressing alrm-Gal4 driven CD8-GFP in just a few cells (see materials and methods). While the 6 glial cells proximal to the dorsal tip are not labeled with mCD8::GFP (close-up of dorsal tip, region 5), four cells that are located further away from the lobe are (see close-ups of cell 1–4). Grey represents FasII and Repo staining (G_1_ and upper panels in close-ups), DAPI staining in the lower panels in close ups or alrm-Gal4 driven CD8-GFP (G_2_ and middle panels in close-ups). Magenta represents FasII and Repo staining and green represents alrm-Gal4 driven CD8-GFP in H_3_ and lower panels in close-ups. The scales bars are 20 μm. Genotypes: (A–E): w;UAS-RedStinger/+;alrm-GAL4/+. (G) y,w,hsFlp,FRT19A/y,w,hsFlp,19A,tubP-GAL80;alrm-GAL4;alrm-GAL4,UAS-mCD8::GFP.

The decrease in alrm positive membrane staining during neuronal remodeling (as depicted in Figure 1) could be due to either a decrease in astrocyte cell number (due to cell death, differentiation or migration) or to a decrease in alrm-Gal4 expression leading to a reduction in CD8-GFP. In order to discriminate between these options we expressed nuclear RFP (RedStinger) driven by alrm-Gal4. While it has been previously shown that there are 6–8 repo-positive cells in the vicinity of each MB lobe during pruning [8], we found only 1–2 astrocytes present at the tip of each γ lobe throughout development (Figure 3A–D, quantified in F). Thus, we found that the number and distribution of astrocytes was largely unchanged throughout development suggesting that the observed lack of glial membranes after 24 h APF is a result of reduced CD8-GFP expression. The reason we could still see RedStinger expression is due to a much stronger and possibly more stable signal, which is characteristic of nuclear transgenes. Thus, astrocytes are a small glia subgroup of 1–2 out of the 6–8 glia within the 5µM vicinity of the lobe (Figure 3E). The identity or the role of the remaining 5–7 repo^+^, alrm^−^ glial cells near the tips remains to be determined. Next we wanted to ask whether these 1–2 astrocytes that are within 5µM of the lobe are the only astrocytes engulfing the degenerating γ lobe. Using sparse labeling (see experimental procedures), we found that distant glia can also send processes to the lobe (Figure 3G). In this example, despite the fact that we could see no labeled glia within the vicinity of the lobe (Figure 3G_1_, 3G_3_, Dorsal Tip 5), the degenerating lobe was still infiltrated by labeled glial membranes (Figure 3G_1_, 3G_3_, green arrows), suggesting that distant glia can also participate in the process of engulfment. Indeed, we found four astrocyte cell bodies that were up to 50µM from the MB lobes but nonetheless sent highly branched processes into the degenerating lobe (Figure 3G, see specific high magnifications of cell 1–4 and Dorsal Tip 5). Therefore, although astrocytes are a small glia sub-population, the effective number of astrocytes that contribute to pruning is more than we initially thought.

While attempting to identify these glia we tested other putative glial drivers and found that mz1127-Gal4, previously shown to specifically mark lamina glia [16], also marked what appeared to be astrocytes (Figure S1E). To determine whether mz1127-Gal4 and alrm-Gal4 labeled the same or potentially a different population of astrocytes, we co-labeled glia with RedStinger driven by both alrm-Gal4 and by mz1127-Gal4 and counted the number of cell bodies in the vicinity of the MB tips at 6 h APF. We found that the number of cells remained 1–2 within 5µM of the lobe tip (Figure S1F and data not shown), suggesting that mz1127-Gal4 expression indeed overlaps with alrm-Gal4 at this developmental time point. This tool might become useful as an additional astrocyte specific driver in the MB.

### Astrocytes are necessary for efficient axon pruning

**Figure 4:**
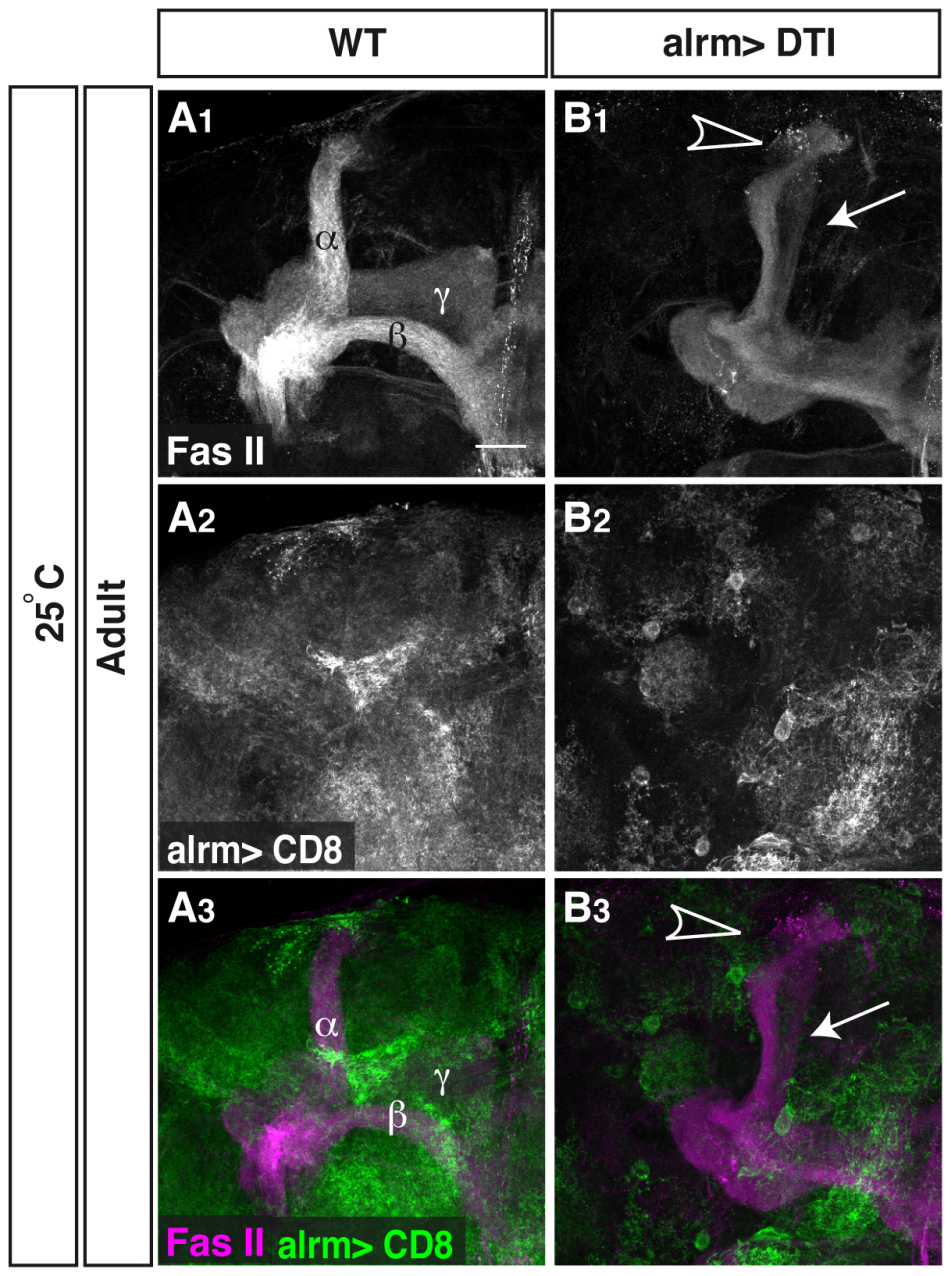
Astrocytes are necessary for efficient axon pruning. (A–B) Confocal Z-projections of adult brains expressing CD8-GFP (A) or additionally UAS-DTI (diphtheria toxin; B) driven by alrm-Gal4. Driving the expression of DTI in astrocytes resulted in their partial ablation (grey, A_2,_ B_2_) and results in fragmentation defects (arrow in B_1_) and uncleared debris (arrowhead in B_1_) in escapers (13 out of 118 expected flies). Grey in A_1_–B_1_ and magenta in A_3_–B_3_ represents anti-FasII. Grey in A_2_–B_2_ and green in A_3_–B_3_ are alrm-GAL4 driven mCD8::GFP. The scale bar is 20 μm. Genotypes: (A) y,w;Sp/CyO;alrm-GAL4,UAS-mCD8::GFP/+; (B) y,w;UAS-DTI/+;alrm GAL4, UAS-mCD8::GFP/+.

In order to further delineate the specific role of astrocytes in axon pruning we decided to completely ablate them from the animal. In order to do so, we expressed the highly toxic diphtheria toxin (DTI) under the control of alrm-Gal4. Unfortunately, when the flies were reared at 29°C, where the expression of the Gal4 is high, this resulted in animal lethality suggesting that astrocytes are essential for normal development. However, when we reared the flies at 25°C to lower the expression level we obtained some escapers who reached adulthood. These flies showed only partial ablation of their astrocytes (compare GFP intensity and distribution in Figure 4A_2_ to 4B_2_). Nonetheless, we detected a mild axon pruning defect in flies with partially ablated astrocytes that manifested in both intact larval γ axons in 70% of the flies (9 out of 13 flies, arrow in Figure 4B) and uncleared axonal debris in 30% of flies (4 out of 13 flies, arrowhead in Figure 4B, compare to 4A). Taken together, these results suggest that astrocytes are necessary for priming γ axons to prune as well as to clear away the debris of fragmented axons.

### Ecdysone receptor in astrocytes regulates multiple aspects of axon pruning

**Figure 5:**
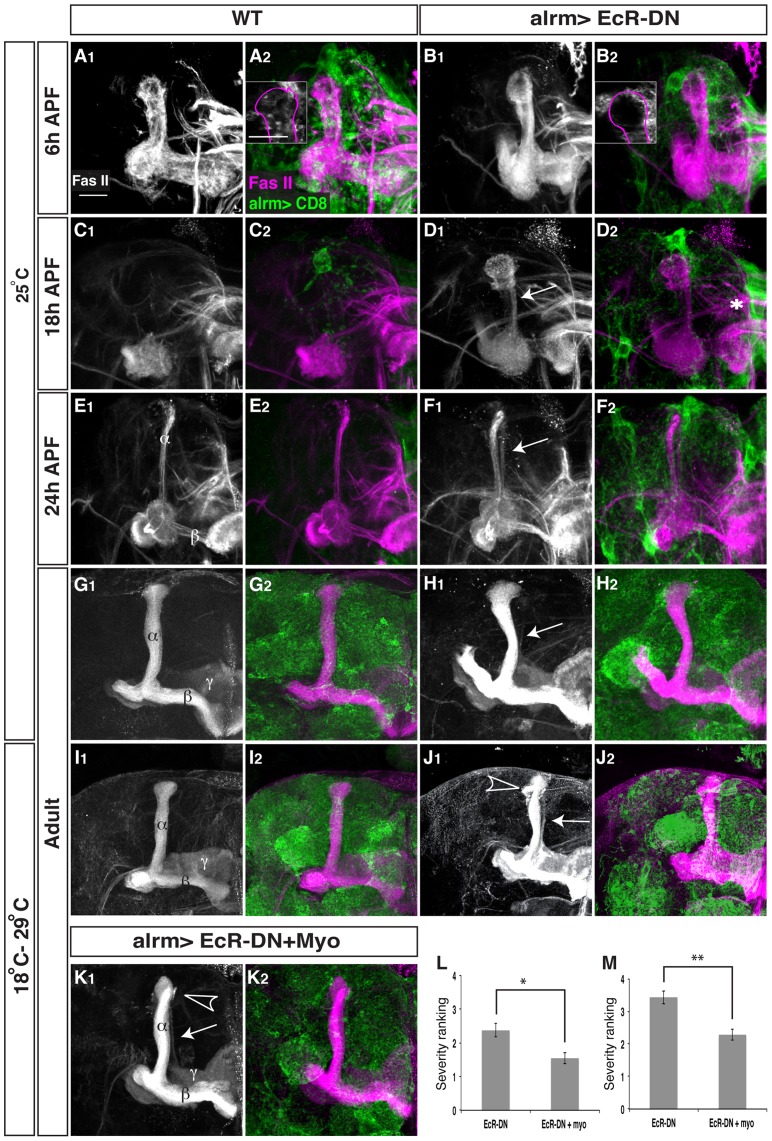
Astrocyte's *Ecdysone Receptor* controls multiply aspects of MB γ neuron remodeling. Confocal Z-projections of brains expressing CD8-GFP driven by alrm-Gal4 (A, C, E, G, I), or those additionally expressing EcR-DN (B, D, F, H, J) or myo in addition to EcR-DN (K) in astrocytes at 6 h APF (A, B), 18 h APF (C, D), 24 h APF (E, F) and Adult (G–K) reared at either 25°C (A–H) or reared at 18°C until late larval stage and then transferred to 29°C until eclosion (see Fig. S2B, I–K). (A,B) At 6 h APF astrocytes expressing EcR-DN do not infiltrate the MB γ lobes (see insets). Overexpression of EcR in astrocytes results in a delay in axon pruning at 18 h APF (D compare to C) and at 24 h APF (F compare to E) that persisted into adult (H compare to G). Elevated expression in astrocytes (I–J; see S2A for details) resulted in more persistent unpruned axons. Overexpression of Myo in addition to EcR-DN (K) partially rescued both the fragmentation (L) and the debris clearance (M) defects. Quantification was performed by ranking analyses that are detailed in the materials and methods section. * P<0.05; ** P<0.005. Bars represent SEM. Grey in A_1_–K_1_ and magenta in A_2_–K_2_ represents anti-FasII staining. Green in A_2_–K_2_ represents alrm-GAL4 driven mCD8::GFP. Scale bar is 20 μM. Genotypes: (A–I): y,w; alrm-GAL4/+;alrm-GAL4,mCD8::GFP/+ (B–J): y,w; alrm-GAL4/UAS-EcR-DN;alrm-GAL4,mCD8::GFP/+. (K): y,w; alrm-GAL4/UAS-EcR-DN;alrm-GAL4,mCD8::GFP/UAS-Myo.

One major determinant of *Drosophila* MB axon pruning is the insect hormone ecdysone [17] that activates the ecdysone receptor B1 (EcR-B1) on γ neurons which then initiates a pruning transcriptional program that functions cell autonomously (Figure 1A) [7]. Such a systemic signal could potentially coordinate between axon pruning and glial function. Thus, we next wanted to see if ecdysone induced signaling also plays a role in astrocytes during MB axon pruning. In order to pursue this, we expressed a dominant negative version of the ecdysone receptor (EcR-DN) specifically in astrocytes and examined their morphology, distribution and any alterations in γ axon pruning. First, we noticed that expressing EcR-DN in astrocytes resulted in changes in their morphology and expression pattern. In WT, at 6 h APF astrocytes have acquired a cyst-like morphology, seem to surround the lobe fully and send small processes that infiltrate the lobe (20 out of 20 examined lobes, Figure 5A_2_, see inset). By 18 h APF there are few GFP positive membranes in the area and they have become much longer and send “finger-like” extensions (Figure 5C_2_). At 24 h APF there is almost no alrm-Gal4 positive membrane staining in the vicinity of the MB, even though the cell bodies remain (Figure 5E_2_, Figure 3D). Finally, by adulthood, the astrocytes seem to obtain the classical astrocyte like morphology and occupy the entire brain (Figure 5G_2_). In contrast, however, when EcR-DN is expressed in astrocytes their morphology is altered: At 6 h APF the astrocytic processes do not infiltrate the MB lobes (22 out of 22 examined lobes, Figure 5B_2_, see inset) and they lack the cyst-like morphology clearly evident in WT astrocytes. At 18 h APF and 24 h APF the astrocytes still have very long processes, and express high levels of CD8-GFP compared to the control (Figure 5D_2_,F_2_, compare with 5C_2_,E_2_). The alterations in astrocyte morphology are not due to changes in the location and/or number of astrocytes since we still see 1–2 cells at each MB lobe tip at these time points (Figure S2A). This suggests that ecdysone signaling is important for priming the astrocytes for their function during pruning.

Next, when we examined γ neurons (by looking at FasII staining), we found that expressing EcR-DN in astrocytes resulted in a significant delay in axon pruning that was apparent at 18 h APF (compare Figure 5D to Figure 5C) and 24 h APF (compare Figure 5F to Figure 5E). At 18APF WT MBs display the initiation of fragmentation (13 out of 18 partially fragmented γ lobes) while some lobes have been completely fragmented at this time point (5 out of 18). In contrast, no MB γ lobes in astrocytic EcR-DN flies show signs of fragmentation (18 out of 18). Later during development, at 24APF, most WT γ lobes have been completely fragmented (13 out of 16) while none were completely fragmented in EcR-DN flies (12 out of 12). In adult, these flies displayed a very weak fragmentation defect (11 out of 16 flies, arrow in Figure 5H, compare to 5G) and sometimes also uncleared debris (data not shown). Due to the weak phenotype observed in the adult MB we decided to raise expression levels of EcR-DN, at the remodeling time window, by rearing the flies at 18°C until late larval stage and then transferring them to 29°C until eclosion (Figure S2B). Indeed, elevated EcR-DN expression resulted in an exacerbation of both the fragmentation defect (9 out of 11 flies, arrow in Figure 5J) and the debris clearance defect (arrowhead in Figure 5J).

Myoglianin (myo) is a *Drosophila* TGF-β ligand secreted by glia that plays a major role in instructing the MB to undergo neuronal remodeling by increasing EcR-B1 levels in the γ neurons, a necessary step in the progression of axon pruning [4]. Myo has been previously shown to be secreted from both cortex glia and astrocytes although it seemed that cortex glia play a more crucial role in myo secretion [4]. Nonetheless, we decided to test whether expression of EcR-DN in astrocytes might affect pruning by affecting myo secretion. We thus overexpressed myo in addition to EcR-DN in astrocytes and found that forced expression of myo partially suppressed both the fragmentation and the debris clearance defects apparent in EcR-DN animals (Figure 5K, quantified in 5L and 5M). UAS-myo by itself had no effect on any MB morphology (Figure S2C). We also investigated whether expressing EcR-DN in astrocytes had an effect on neuronal expression of EcR-B1 at 0APF as predicted if myo secretion was indeed diminished. To our surprise, we found no such reduction in neuronal EcR-B1 levels (Figure S2D) suggesting that although myo seems to be important for the function of astrocytes, the EcR-DN induced phenotype in astrocytes is not likely to stem solely from insufficient myo dependent induction of EcR-B1 in neurons. It will be interesting to further delineate the EcR dependent program within astrocytes that is responsible for their morphological change and their ability to promote γ neuron pruning.

### Astrocytes engulf MB γ axon debris via Draper

**Figure 6:**
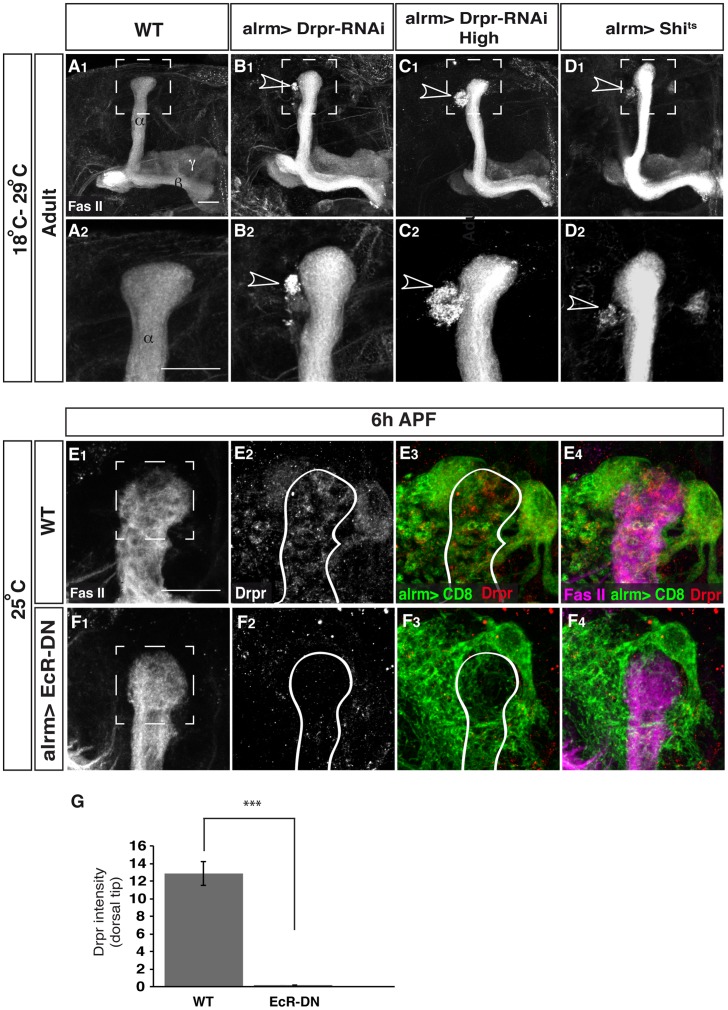
Knocking down the ecdysone regulated *drpr* or inhibiting endocytosis in astrocytes results in uncleared axonal debris. Confocal Z-projections of adult (A–D) or 6APF brains (E, F) expressing CD8-GFP (A, E), additionally expressing Drpr-RNAi (B, C), *Shi^ts1^* (D), or EcR-DN (F) in astrocytes using the alrm-Gal4 driver. High and prolonged expression of Drpr-RNAi (C) was achieved by the Tub>GAL4 flipout system (see Fig. S3A for details) (A_2_–D_2_) Represent a higher-magnification of the dorsal tip. No ectopic γ axon branches were detected in adults when astrocytic engulfment ability was impaired with expression of *Drpr* RNAi (B_1_, C_1_) or *shibire^ts^* (D_1_). Uncleared debris (arrowhead) was detected in 100% of flies of the tested transgenes (*Drpr* RNAi, 12 flies, B_2_; forced expressed *Drpr* RNAi, 12 flies, C_2_ and *shibire^ts^*, 17 flies, D_2_). (E, F) Drpr staining in WT (E) and flies expressing EcR-DN in astrocytes (F) at 6APF. Marked box represents regions that were used fort the quantification in G. (G) EcR-DN expressing flies show significantly lower staining for Drpr (0.16±0.01, n = 14) than WT flies (12.88±1.37, n = 10, p<0.001) at 6APF (quantification was done on confocal single slices). Grey represent anti-FasII staining in A–D and in E_1_–F_1_ and Drpr staining in E_2_–F_2_. Green in E and F represents alrm-GAL4 driven mCD8::GFP. Red in E_3–4_ and F_3–4_ is Drpr staining. The scale bars are 20 μm. Genotypes: (A) y,w;alrm-GAL4/+;alrm-GAL4,mCD8::GFP/+; (B) y,w;alrm-GAL4/+;alrm-GAL4,mCD8::GFP,Dcr2/UAS-Drpr-RNAi; (C) y,w;P{GAL4-αTub84B(FRT.CD2).P}/UAS-FLP;alrm-GAL4,mCD8::GFP,Dcr2/UAS-Drpr-RNA (D) y,w;alrm-GAL4/+;alrm-GAL4,mCD8::GFP/UAS-Shit^ts^. (E) y,w; alrm-GAL4/+;alrm-GAL4,mCD8::GFP/+ (F) y,w; alrm-GAL4/UAS-EcR-DN;alrm-GAL4,mCD8::GFP/+.

Previous work has shown that the cell corpse engulfment receptor Draper (Drpr) is expressed in glial cells and is necessary for the engulfment of γ axon fragments following pruning [10]. We next wished to see if a) the engulfment of γ axons by astrocytes is also mediated by Drpr and b) if engulfment by astrocytes is active or passive. To do so we expressed Drpr RNAi in astrocytes and indeed saw a defect in debris clearance but not in the fragmentation process itself, as we could not see intact larval axons (Figure 6B, compare to Figure 6A). The lack of effect on axon fragmentation could be a real biological finding but could, in theory, result from low expression level of the RNAi (as we have already shown that alrm-Gal4 levels go down at 18 h and 24 h APF). Therefore, we decided to increase and prolong the expression of the RNAi by using a flip out tubulin-Gal4 system [18](Figure S3). While this increased expression of Drpr RNAi did exacerbate the amount of uncleared debris (Figure 6C), we still never observed a defect in axon fragmentation. This indicates that while Drpr does play a role in engulfing fragmented axons, it is a permissive rather than instructive role. Further support comes from the finding that disrupting endocytosis by expressing a dominant negative temperature-sensitive version of dynamin (called shibire in *Drosophila*; UAS-shi^ts^) in astrocytes also only caused a defect in debris clearance without affecting axon fragmentation (Figure 6D).

Because we have shown earlier that the ecdysone receptor is cell-autonomously required in glia for both induction of axon fragmentation and engulfment of axonal debris, we decided to examine whether Drpr expression might be regulated by ecdysone signaling. To this end, we examined if there were alterations in the expression of Drpr in animals expressing EcR-DN in astrocytes. Indeed, while WT astrocytes express high amounts of Drpr at 6APF (average intensity of 12.88±1.37 in the immediate vicinity of the dorsal lobe, n = 10, Figure 6E, quantification in 6G), EcR-DN expressing astrocytes show substantially lower expression (average intensity of 0.16±0.01, p<0.001 compared to WT, Figure 6F, quantification in 6G). Taken together, it appears that astrocytes have two distinct roles: first, they instruct γ neuron fragmentation in an ecdysone dependent pathway and second they are the major glial type that clears axonal debris in a pathway that is also mediated by ecdysone signaling which increases Drpr expression and by endocytosis.

## Discussion

The role of glia in shaping the nervous system has gained much traction in the past few years [19]. In this study we further dissected the involvement of glia in the neuronal remodeling of the *Drosophila* MB. We show that astrocytes are one of the major glial subtypes that is responsible for MB γ axon pruning, despite the fact that they make up a small subpopulation of glia surrounding the MB during this developmental period. We also show that astrocytes' morphological maturation, promotion of axon fragmentation and debris engulfment depend on glial cell autonomous ecdysone receptor signaling. Furthermore, we can block the ability of astrocytes to clear MB axonal debris, but not affect their morphological change or ability to promote axon fragmentation, by blocking endocytosis or knocking down the expression of the engulfment receptor Drpr, thus decoupling these two processes. We therefore propose that astrocytes play a key role in both promoting axon fragmentation as well as clearing axonal debris once fragmentation has been completed.

### Astrocytes play a key role in debris clearance during MB axon pruning

Previous work has shown that glia participate in axon pruning by playing an important role in the clearance of neuronal debris following γ axon fragmentation. This was shown to depend on the engulfment receptor Drpr and on glial endocytosis [8], [9] suggesting that axonal debris is degraded by the endosome-lysosomal pathway [8] although the exact nature of these glia remained unknown. Here we show that astrocytes are the glial subtype that is responsible for most, if not all, of the infiltration and engulfment of neuronal debris following fragmentation. This was surprising for two major reasons. One is that ensheathing glia have already been shown to act as phagocytes, albeit in the adult brain [12]. Another reason these results were surprising is that astrocytes make up only a minority of the glia surrounding the MB lobes during pruning, comprising of only 1–2 astrocytes out of 6–8 total glia in the vicinity of each lobe. However, using two binary systems to simultaneously label neurons and astrocytes, we demonstrated that most, if not all, axonal debris is engulfed by astrocytes. Furthermore, although there are only 1–2 astrocytic cell bodies located near each tip, we found that cells that are located further away, up to 50µm, can also infiltrate the degenerating lobe, thereby potentially participating in debris clearance.

The identity of the remaining 4–7 alrm^−^ repo^+^ glia surrounding the tips during MB neuronal remodeling remains unknown. Dissecting the nature of these glia and how they contribute to axon fragmentation and elimination remains to be further investigated.

### Ecdysone receptor controls astrocyte function during pruning

One of the major determinants of metamorphosis and of axon pruning is the insect hormone ecdysone. Ecdysone receptor B1 (EcR-B1) is cell autonomously required in MB γ axons to initiate a transcriptional program that, in turn, mediates axon pruning [7]. EcR-B1 is also necessary for the dendrite pruning of dendritic arborization (DA) neurons [20] as well as for the remodeling of embryonic olfactory projection neurons [21]. In addition, ecdysone plays a role in the refinement of the fly neuromuscular junction [22]. As all these neuronal remodeling processes occur in the pupae, this raises the hypothesis that ecdysone may act as a master regulator of multiple remodeling processes. In addition, ecdysone being a systemic hormone, can function as a potential coordinator between fragmentation in neurons with phagocytic cells. Although the effect of ecdysone on neurons has been extensively studied [7], [20]–[23], the effects of ecdysone on the surrounding glia have remained largely unknown.

To further investigate the role of astrocytes in MB axon pruning, we expressed a dominant negative version of the ecdysone receptor (EcR-DN) specifically in astrocytes and discovered defects in glial morphology, γ axon fragmentation and axonal debris clearance. The defect in debris clearance is, at least partially, due to decreased expression of the cell corpse engulfment receptor Drpr. This is consistent with previous results showing that expression of EcR-DN in glia leads to a defect in Drpr transcript upregulation following metamorphosis [10].

Up to this point, the only instructive role that has been shown for glia in the neuronal remodeling of the MB γ neurons has been through the secretion of myoglianin (myo), leading to an increase in γ neuron EcR-B1 levels [4]. Myo secretion has been shown to be crucial in cortex glia and less so in astrocytes [4]. We found that expression of EcR-DN in astrocytes, resulted in a partial pruning defect, manifested as intact larval axons as well as decreased axonal debris clearance, with no evident decrease in EcR-B1 expression in MB γ neurons. Furthermore, we found that forced expression of myo in addition to EcR-DN in astrocytes only partially rescues the pruning defect of MB γ neurons. This suggests that the pruning defect apparent in these adult flies is not mediated solely by a decrease in myo secretion but that there is an additional mechanism at play. This might involve astrocytes morphology and distribution, which was previously shown to be important for proper neuronal circuit development in other systems. For instance, in autistic subjects astrocytes exhibit significantly reduced branching processes, total branching length and cell body sizes [24]. In the wobbler mouse, which displays a muscular atrophy associated with motoneuron degeneration in early postnatal development, the glial fibrillary acidic protein (GFAP) is greatly increased in the spinal cord resulting in many changes in the number, distribution and morphology of astrocytes [25]. Thus, the ecdysone receptor dependent program in astrocytes resulting in morphological changes and pruning promotion remains to be fully delineated. This signaling cascade is especially interesting since very few downstream targets of ecdysone are known in any process of neuronal remodeling. For example, while Sox-14 is an important ecdysone induced effector in DA dendrite pruning [20], its requirement in MB remodeling remains to be further examined. Thus, delineating ecdysone signaling in both glia and MB neurons during pruning requires further investigation.

The apoptotic cell engulfment receptor Drpr is known to play a key role in mediating glial clearance of severed axons following injury [26] and during developmental axon pruning [10] however its role in astrocytes has not been investigated. We found that expressing high levels of Drpr RNAi or disrupting endocytosis in astrocytes disrupted axon debris clearance but, in contrast to the EcR pathway, did not cause a defect in axon fragmentation. Furthermore, EcR signaling appears to regulate Drpr expression implicating Drpr as one of the ecdysone induced effectors in MB remodeling.

It appears that ecdysone acts as a master regulator in astrocytes to regulate multiple functions during remodeling. These include changing astrocyte morphology, instructing γ neurons to undergo axon fragmentation and clearing axonal debris after fragmentation. In contrast Drpr and the endocytotic pathway mediate only debris clearance. Thus, it will be of great interest to delineate the EcR dependent and additional molecular mechanisms that control astrocyte morphology and function during neuronal remodeling.

During the review process of this manuscript two studies have been published that further support the role of astrocytes during remodeling. In Chung et al [27], astrocytes were shown to play a crucial role in the elimination of mammalian synapses via phagocytosis. Taşdemir-Yilmaz at al [28] show that astrocytes are important for engulfment of neuronal debris during neuronal remodeling in *Drosophila*, of both the MB and vCrz positive neurons in the ventral nerve cord. These two recent studies, together with ours, place astrocytes as a major phagocytic glial type during development of the nervous system of vertebrates and invertebrates.

## Experimental Procedures

### Drosophila Strains

*Drosophila* strains used: *UAS-EcR.B-ΔC655.W650A*; *P{M2ET-QF}ET40*; *P{QUAS-mtdTomato-3xHA}14*; *P{UAS-RedStinger}*; *Drpr-RNAi*; *P{UAS-FLP.Exel}2* and *P{UAS-Cbβ\DT-A.I}18* were obtained from Bloomington *Drosophila* stock center (Indiana University, USA).

*Tub>CD2>Gal4* was a kind gift from F. Pignoni, Upstate Medical University, Syracuse, NY; *alrm*-*Gal4* and *mZ0709-Gal4* were kindly provided by M. Freeman, UMass, Worcester, MA; UAS-myo was kindly provided by T. Lee, Janelia Farm, HHMI, Virginia; *mZ1127-GAL4* was a kind gift from I. Salecker, National Institute for Medical Research, London; UAS-*Shi^ts1-pJFRC100^* was provided by GM Rubin, Janelia Farm, HHMI, Virginia.

### Antibody Staining Conditions

Brains were dissected and stained as previously described [29] and imaged on a Zeiss LSM710 confocal microscope.

Antibodies were used at the following dilutions:

Rat monoclonal anti-mouse CD8 α subunit, 1∶200 (Caltag, Burlingame, CA, USA), Chicken anti-GFP, 1∶500; Rabbit anti-HA (H6908), 1∶100 (Sigma-Aldrich, Saint-Louis, MO, USA); The remaining antibodies were all obtained from the Developmental Studies Hybridoma Bank: mouse monoclonal anti-FasII (1D4), 1∶50; mouse monoclonal anti-EcR-B1 (AD4.4), 1∶25 mouse monoclonal anti-Repo (8D1.2), 1∶10; mouse monoclonal anti-Draper (8A1), 1∶400. Alexa 488, Alexa546 or Alexa633 conjugated secondary antibodies were used at 1∶300 (Invitrogen). DAPI (4′,6-Diamidino-2-Phenylindole, Dihydrochloride) 1∶1000. In order to stain for two anti-mouse antibodies concurrently we used Zenon (Invitrogen).

### Fly genetics

*Drosophila melanogaster* were grown on standard media at 25°C or at indicated temperatures to manipulate Gal4 expression.

To achieve sparse labeling of astrocytes, we aimed at performing a MARCM experiment. However, we found that our MARCM fly, containing alrm-Gal4, UAS-CD8-GFP and tubP-Gal80 expressed CD8-GFP in a few (seemingly random) cells.

### Glia and astrocytes nuclei quantification

Glia and astrocytes cell number were quantified by counting the number of glial nuclei, as labeled with anti-Repo antibody and the nuclear RFP reporter (RedStinger) driven by alrm-Gal4, respectively. Confocal Z stacks were used as three-dimensional representations of the MB environment. To quantify the number of cells in the immediate vicinity of the MB lobe, we counted nuclei that were within 5 µM of the designated area (as illustrated in Figure 3E).

### EcR-DN and Myo forced expression in astrocytes pruning and debris clearance severity comparison

To analyze the severity of the pruning and debris clearance phenotype 18 and 20 confocal LSM files of EcR-DN and Myo forced expressed with EcR-DN, respectively, were blindly ranked from 0 (WT) to 5 (most severe pruning defect/debris clearance defect) by 4 independent researchers. Pruning severity was determined by comparing unpruned axonal branches that are significantly outside the α lobe, which is defined by strong FasII immunoreactivity. Analysis was done using Statistica software using repeated measures ANOVA.

### Quantification of Draper expression levels

Drpr antibody staining was examined in the immediate vicinity of the dorsal γ lobe. Quantification of intensity in confocal single slices was performed using ImageJ software. Levels of Drpr in WT and EcR-DN expressing animals were compared using student's T-test.

## Supporting Information

Figure S1 **Characterization of glial subtypes** ***GAL4*** **drivers,** ***MZ0709- GAL4*** **and** ***MZ1127-GAL4*** **(refers to** **figure 1** **and** **3** **).** Confocal Z-projections of brains expressing CD8-GFP driven by *MZ0709-GAL4* (A-D) and *MZ1127-GAL4* (E–F) in the mushroom body (MB; A,B,E,F) or antenna lobe (AL; C,D) at either the adult stage (A,B) or at 6 APF (C–F). (A,C) At the adult stage, mCD8::GFP driven by the ensheathing glia driver, *MZ0709-GAL4*, was co-localized with the glial nuclei marker Repo (magenta) in both the MB and the AL (A and C, arrows). (B, D) At 6APF there was no co-localization of CD8 and repo neither in the MB (B) nor at the AL (D) indicating that this driver cannot be used to study glia-neuron interaction during pruning. (E) *MZ1127-GAL4* driven mCD8::GFP at 6APF labeled a glial subtype that is reminiscent of astrocyte morphology. (F) Nuclear reporter (UAS-RedStinger) was driven with both *alrm-GAL4* and *MZ1127-GAL4*. In all the brains (9 flies) 1–2 nuclei were stained near the dorsal lobe tip (F) as was found previously when alrm-GAL4 was expressed by itself (Fig. 3F). Magenta in A–D represent anti-Repo and in E–F anti-Fas II and anti-Repo. Green in A–D is mz0907-GAL4 and in E mz1127-GAL4 driven mCD8::GFP. Red in A and B is QF-ET40 driven QUAS-mtdt-3xHA and in F alrm- and mz1127-Gal4s driving RedStinger expression. The scale bars are 20 μm. Genotypes: (A–D) y,w;QF-ET40,QUAS-mtdt-3xHA/CyO;MZ0709-GAL4,UAS-mCD8::GFP/+ (E) y,w;mz1127-GAL4,UAS-mCD8::GFP (F) y,w;UAS-RedStinger/mz1127-GAL4;alrm-GAL4/+.
(TIF)

Figure S2 **EcR-DN and UAS-Myo expressed in astrocytes (refers to** **figure 5** **).** (A) Quantification of the number of astrocytes nuclei in brains expressing EcR-DN driven by Alrm-Gal4. (B) A schematic illustration of the rearing regime of flies reared at 18°C until late larval stage and then transferred to 29°C until eclosion. (C) No defect in axon fragmentation or uncleared debris were detected when UAS-Myo was expressed in astrocytes of flies reared at 18°C until late larval stage and then transferred to 29°C until eclosion. (D) Overexpression of myo in addition to EcR-DN in astrocytes did not significantly suppress the defects of EcR-DN expression. Grey and magenta represent anti-Fas II. Green is alrm-GAL4 driven mCD8::GFP. (D) EcR-B1 expression levels in MB Kenyon cells (illustration D_1_) are not affected by EcR-DN expressed in astrocytes (compare D_2_–D_3_). Grey represents anti-EcR B1. Scale bars are 20 μm. Genotypes (C) y,w; alrm-GAL4/+;alrm-GAL4,mCD8::GFP/UAS-Myo (D) y,w; alrm-GAL4/UAS-EcR-DN;alrm-GAL4,mCD8::GFP/+.
(TIF)

Figure S3 **Schematic illustration of the molecular mechanisms of the Tub>GAL4 flip out system forced expresses Drpr-RNAi.** (A) alrm-GAL4 activates the expression of Drpr-RNAi and FLP recombinase in astrocytes. Astrocytes expressing FLP recombinase subsequently excise the FRT-flanked CD2 cassette separating the Tubulin promoter and GAL4 open reading frame. This maintains a strong and from now on also alrm independent expression of Drpr-RNAi in the astrocytes. Genotype: y,w;P{GAL4-αTub84B(FRT.CD2).P}/UAS-FLP;alrm-GAL4,mCD8::GFP/UAS-Drpr-RNA.
(TIF)
