# Supplementary figures and images for: Astrocytes Play a Key Role in Drosophila Mushroom Body Axon Pruning
Source: PLoS One. 2014 Jan 21;9(1):e86178. doi: 10.1371/journal.pone.0086178 (PMC3897647; doi:10.1371/journal.pone.0086178)

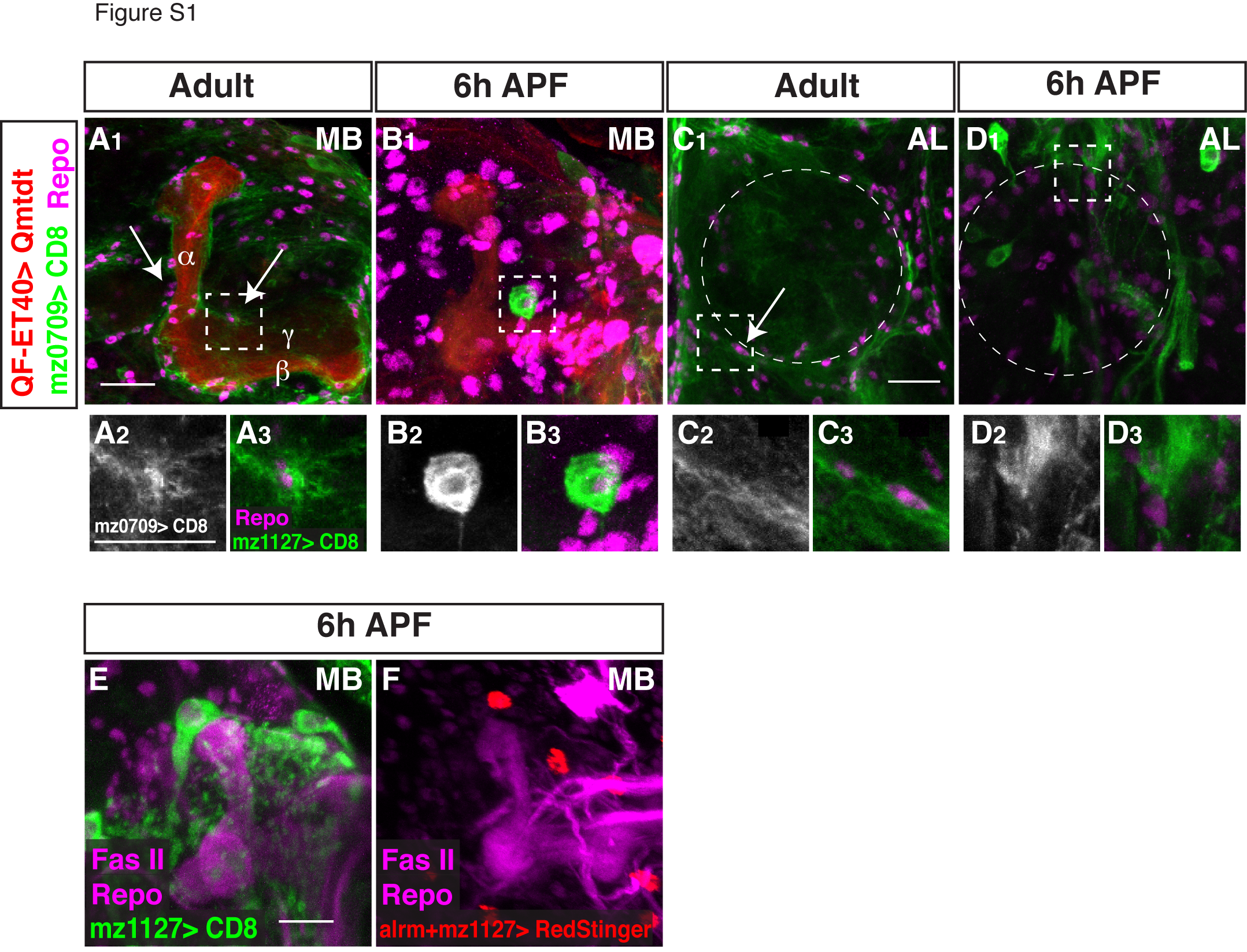

Supplement: Figure S1 — Characterization of glial subtypes GAL4 drivers, MZ0709- GAL4 and MZ1127-GAL4 (refers to figure 1 and 3 ). Confocal Z-projections of brains expressing CD8-GFP driven by MZ0709-GAL4 (A-D) and MZ1127-GAL4 (E–F) in the mushroom body (MB; A,B,E,F) or antenna lobe (AL; C,D) at either the adult stage (A,B) or at 6 APF (C–F). (A,C) At the adult stage, mCD8::GFP driven by the ensheathing glia driver, MZ0709-GAL4, was co-localized with the glial nuclei marker Repo (magenta) in both the MB and the AL (A and C, arrows). (B, D) At 6APF there was no co-localization of CD8 and repo neither in the MB (B) nor at the AL (D) indicating that this driver cannot be used to study glia-neuron interaction during pruning. (E) MZ1127-GAL4 driven mCD8::GFP at 6APF labeled a glial subtype that is reminiscent of astrocyte morphology. (F) Nuclear reporter (UAS-RedStinger) was driven with both alrm-GAL4 and MZ1127-GAL4. In all the brains (9 flies) 1–2 nuclei were stained near the dorsal lobe tip (F) as was found previously when alrm-GAL4 was expressed by itself (Fig. 3F). Magenta in A–D represent anti-Repo and in E–F anti-Fas II and anti-Repo. Green in A–D is mz0907-GAL4 and in E mz1127-GAL4 driven mCD8::GFP. Red in A and B is QF-ET40 driven QUAS-mtdt-3xHA and in F alrm- and mz1127-Gal4s driving RedStinger expression. The scale bars are 20 μm. Genotypes: (A–D) y,w;QF-ET40,QUAS-mtdt-3xHA/CyO;MZ0709-GAL4,UAS-mCD8::GFP/+ (E) y,w;mz1127-GAL4,UAS-mCD8::GFP (F) y,w;UAS-RedStinger/mz1127-GAL4;alrm-GAL4/+. (TIF) [file pone.0086178.s001.tif]

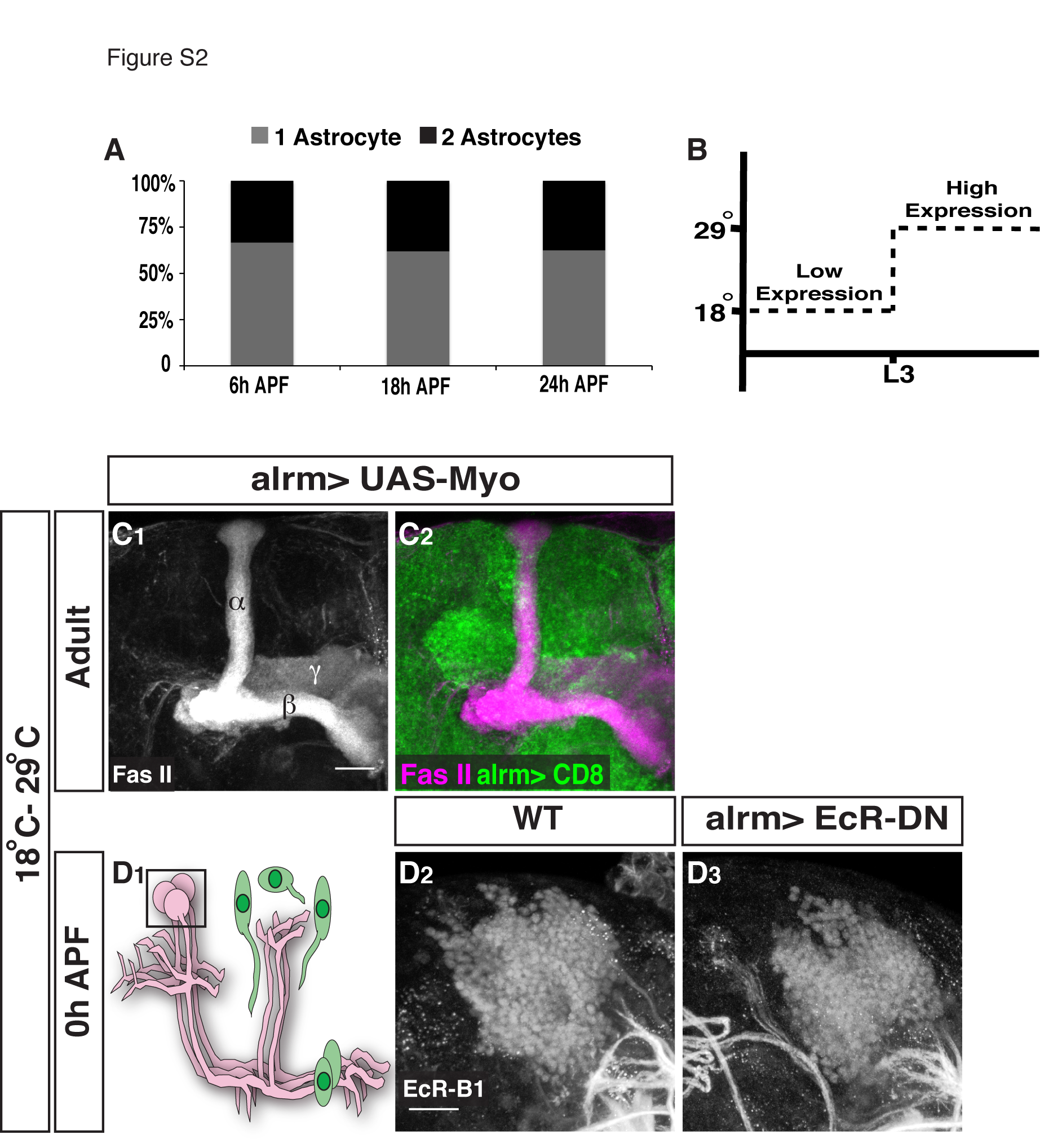

Supplement: Figure S2 — EcR-DN and UAS-Myo expressed in astrocytes (refers to figure 5 ). (A) Quantification of the number of astrocytes nuclei in brains expressing EcR-DN driven by Alrm-Gal4. (B) A schematic illustration of the rearing regime of flies reared at 18°C until late larval stage and then transferred to 29°C until eclosion. (C) No defect in axon fragmentation or uncleared debris were detected when UAS-Myo was expressed in astrocytes of flies reared at 18°C until late larval stage and then transferred to 29°C until eclosion. (D) Overexpression of myo in addition to EcR-DN in astrocytes did not significantly suppress the defects of EcR-DN expression. Grey and magenta represent anti-Fas II. Green is alrm-GAL4 driven mCD8::GFP. (D) EcR-B1 expression levels in MB Kenyon cells (illustration D1) are not affected by EcR-DN expressed in astrocytes (compare D2–D3). Grey represents anti-EcR B1. Scale bars are 20 μm. Genotypes (C) y,w; alrm-GAL4/+;alrm-GAL4,mCD8::GFP/UAS-Myo (D) y,w; alrm-GAL4/UAS-EcR-DN;alrm-GAL4,mCD8::GFP/+. (TIF) [file pone.0086178.s002.tif]

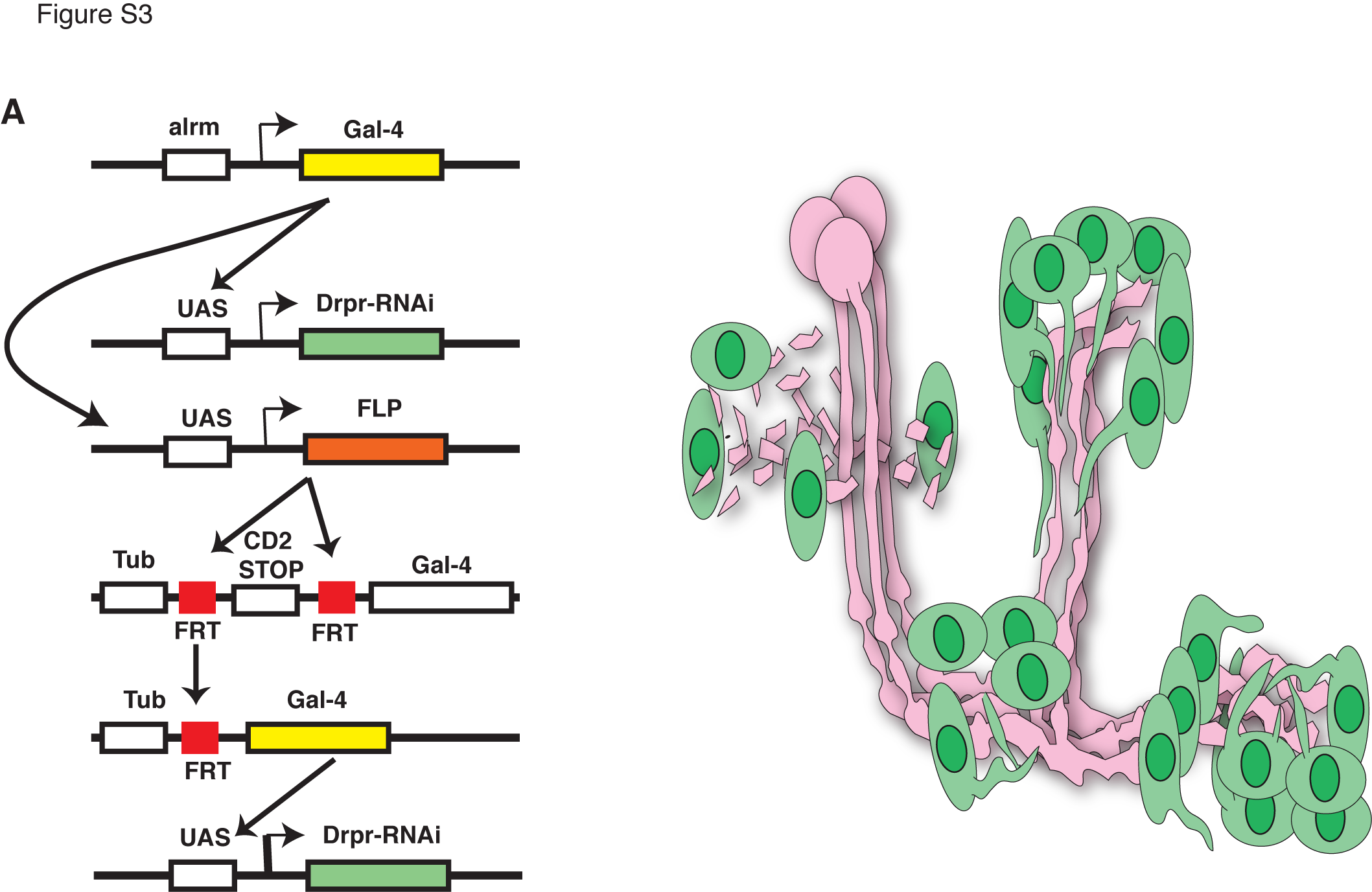

Supplement: Figure S3 — Schematic illustration of the molecular mechanisms of the Tub>GAL4 flip out system forced expresses Drpr-RNAi. (A) alrm-GAL4 activates the expression of Drpr-RNAi and FLP recombinase in astrocytes. Astrocytes expressing FLP recombinase subsequently excise the FRT-flanked CD2 cassette separating the Tubulin promoter and GAL4 open reading frame. This maintains a strong and from now on also alrm independent expression of Drpr-RNAi in the astrocytes. Genotype: y,w;P{GAL4-αTub84B(FRT.CD2).P}/UAS-FLP;alrm-GAL4,mCD8::GFP/UAS-Drpr-RNA. (TIF) [file pone.0086178.s003.tif]
